# Recent recommendations from ATA guidelines to define the upper reference range for serum TSH in the first trimester match reference ranges for pregnant women in Rio de Janeiro

**DOI:** 10.20945/2359-3997000000064

**Published:** 2018-08-01

**Authors:** Nathalie Anne de Oliveira e Silva de Morais, Annie Schtscherbyna Almeida de Assis, Carolina Martins Corcino, Débora Ayres Saraiva, Tatiana Martins Benvenuto Louro Berbara, Carolina Donner de Drummond Ventura, Mario Vaisman, Patrícia de Fátima dos Santos Teixeira

**Affiliations:** 1 Universidade Federal do Rio de Janeiro Universidade Federal do Rio de Janeiro Hospital Universitário Clementino Fraga Filho Faculdade de Medicina Rio de Janeiro RJ Brasil Departamento de Medicina Interna e Unidade de Endocrinología, Faculdade de Medicina e Hospital Universitário Clementino Fraga Filho, Universidade Federal do Rio de Janeiro (HUCFF/UFRJ), Rio de Janeiro, RJ, Brasil; 2 Instituto Estadual de Diabetes e Endocrinologia Luiz Capriglione Unidade de Endocrinologia Rio de Janeiro RJ Brasil Unidade de Endocrinologia, Instituto Estadual de Diabetes e Endocrinologia Luiz Capriglione (IEDE), Rio de Janeiro, RJ, Brasil; 3 Instituto Nacional do Câncer Instituto Nacional de Câncer José Alencar Gomes da Silva Unidade de Endocrinologia Rio de Janeiro RJ Brasil Unidade de Endocrinologia, Instituto Nacional de Câncer José Alencar Gomes da Silva (INCA), Rio de Janeiro, RJ, Brasil

**Keywords:** TSH, pregnancy, reference range

## Abstract

**Objectives:**

American Thyroid Association (ATA)'s new guidelines recommend use of population-based trimester-specific reference range (RR) for thyrotropin (TSH) in pregnancy. The aim of this study was to determine first trimester TSH RR for a population of pregnant women in Rio de Janeiro State.

**Subjects and methods:**

Two hundred and seventy pregnant women without thyroid illness, defined by National Academy of Clinical Biochemistry, and normal iodine status were included in this sectional study. This reference group (RG) had normal median urinary iodine concentration (UIC = 219 μg/L) and negative anti-thyroperoxidase antibodies (TPOAb). Twin pregnancy, trophoblastic disease and use of drugs or supplements that influence thyroid function were excluded. In a second step, we defined a more selective reference group (SRG, n = 170) by excluding patients with thyroiditis pattern on thyroid ultrasound and positive anti-thyroglobulin antibodies. This group also had normal median UIC. At a final step, a more selective reference group (MSRG, n = 130) was defined by excluding any pregnant women with UIC < 150 μg/L.

**Results:**

In the RG, median, 2.5^th^ and 97.5^th^ percentiles of TSH were 1.3, 0.1, and 4.4 mIU/L, respectively. The mean age was 270 ± 5.0 and the mean body mass index was 25.6 ± 5.2 kg/m^2^. In the SRG and MSRG, 2.5^th^ and 975^th^ percentiles were 0.06 and 4.0 (SRG) and 0.1 and 3.6 mIU/L (MSRG), respectively.

**Conclusions:**

In the population studied,TSH upper limit in the first trimester of pregnancy was above 2.5 mIU/L. The value of 3.6 mIU/L, found when iodine deficiency and thyroiditis (defined by antibodies and ultrasound characteristics) were excluded, matches recent ATA guidelines.

## INTRODUCTION

The American Thyroid Association (ATA) has recently published new guidelines for the diagnosis and management of thyroid disease during pregnancy and the postpartum period (1). A substantial difference in these guidelines, compared with the prior version published in 2011 (2), is the question regarding the upper reference limit for serum thyrotropin (TSH) during the first trimester of pregnancy, defined previously as 2.5 mIU/L. ATA's new guidelines recommend the use of population-based trimester-specific reference ranges for serum TSH derived from evaluation of local population data. When local assessments are not available, the panel recommends that lower reference range of TSH should be reduced by approximately 0.4 mIU/L, while the upper reference range should be reduced by approximately 0.5 mIU/L in the first trimester of pregnancy, from the reference values for non-pregnant women (1).

These cutoff changes were based on recent large cohorts from various countries that have published trimester-specific pregnancy reference ranges, demonstrating significantly higher serum TSH concentration in their populations than previously reported in the literature (3-9). Two of these studies showed, by week-to-week evaluation, that TSH reference limits were variable during the first trimester. In their studied populations, median TSH from up to 6 weeks was much higher, similar to the reference range for non-pregnant women, than to the range for 7 to 12 weeks (3,4). This larger analysis also demonstrated substantial population differences in the TSH upper-reference limit that can be explained by differences in ethnicity, geographic location, iodine intake, body mass index (BMI), thyroid peroxidase antibody (TPOAb)-positivity and the TSH assays used for analysis (10).

Recently, there have been published data concerning the normal range of serum TSH for the first trimester of gestation in a non-coastal area of Brazil (11). In the studied population, the TSH value corresponding to the 97.5^th^ percentile was 2.68 mIU/L in the first trimester of gestation, which matches prior guidelines recommendations (2,12,13). In contrast, another smaller Brazilian study, which evaluated pregnant women in the first trimester in a coastal area of the country, found higher TSH levels with a 97.5^th^ percentile of 4.43 mIU/L (14).

The National Academy of Clinical Biochemistry (NACB) states that “TSH reference intervals should be established from 95% confidence limits of the log-transformed values of at least 120 rigorously screened normal euthyroid volunteers who meet the following criteria: no detectable thyroid autoantibodies (TPOAb or thyroglobulin antibody (TgAb), measured by sensitive immunoassay); no personal or family history of thyroid dysfunction; no visible or palpable goiter; and not using any medications (except estrogen)” (15). It is worth mentioning that NACB does not require thyroid ultrasonography to exclude thyroiditis, and that the presence of only one negative thyroid autoantibodies is necessary. The lower and upper limits of TSH are defined by the 2.5^th^ and 97.5^th^ percentile, respectively (16).

In recent years, several studies have shown important additive effects of thyroid autoimmunity on maternal thyroid status with respect to pregnancy outcome. It turns out that women who are antibody positive have a greater risk of adverse events than do antibody-negative women, even when they have identical thyroid function (17,18). There are patients with undetectable thyroid antibodies who may have a thyroiditis pattern on ultrasound (19,20). Furthermore, population studies in non-pregnant women confirm that upper reference range of TSH is reduced after excluding those with ultrasound patterns compatible with thyroiditis (20-22). International guidelines recommend populations with adequate iodine intake to be used to define TSH reference ranges. This is especially important in pregnant populations due to increased iodine demand during pregnancy (1,12,13). Not only iodine deficiency but also high iodine intake is associated with reduced thyroid function (23).

From these data, we proposed this study aiming to establish a serum TSH reference range in the first trimester of gestation for a population that lives in the coastal area of the state of Rio de Janeiro. For this purpose, we assessed a sample of pregnant women without thyroid illness, defined by NACB strict criteria, normal ultrasound patterns, normal iodine status and absence of thyroid autoantibodies (TPOAb and TgAb).

## SUBJECTS AND METHODS

This cross-sectional study was performed on a subpopulation from an ongoing prospective cohort, and included pregnant women attending prenatal programs in maternity clinics in the public health system in the State of Rio de Janeiro. Four prenatal outpatient clinics in a coastal area of the state of Rio de Janeiro, Brazil, participated in this study. The study was approved by the local Research Ethics Committee, and all subjects signed consent forms (CAAE: 22546213.0.0000.5275).

A total of 270 pregnant women were enrolled from September 2014 to January 2017. The recruiting criteria included age ≥ 18 and ≤ 35 years old, having a spontaneous pregnancy and gestational age up to 12 weeks (defined by last menstrual period or ultrasound). According to Guideline 22 of the National Academy of Clinical Biochemistry (15), we excluded patients with personal or familiar history of thyroid disease, using drugs or supplements that might influence thyroid function, and with visible or palpable goiter. Women with multiple pregnancies, trophoblastic disease and other chronic diseases history were also excluded.

Specific history and general physical examination were performed. Height and weight were measured with participants barefoot and wearing light clothing. Body mass index (BMI) was calculated as weight (kg) divided by height squared (m^2^).

Samples of blood and spot urines were obtained from each participant in the morning, after 10 to 12 hours of fasting, to determine serum TSH, FT4, TPOAb, TgAb and urinary iodine concentration (UIC). Thyroid ultrasound scan was performed in all participants.

After this analysis, we excluded patients with positive TPOAb to create the reference group.

This final sample, defined as reference group (RG), consisted of 225 pregnant women that filled all NACB criteria and had a normal median UIC.

In a second step, we defined a selective reference group (SRG, n = 170) by excluding those patients with thyroiditis pattern on thyroid ultrasound scan and positive serum TgAb. This group also had normal median UIC.

Ultrasound thyroiditis pattern was defined as the presence of a heterogeneous and/or hypoechoic gland. Classification of iodine status was made according to the World Health Organization (WHO) as follows: severe insufficiency, < 50 μg/L; mild-moderate insufficiency, 50 to 149 μg/L; sufficiency, 150 to 249 μg/L; more than adequate, 250 to 499 μg/L; and excessive, ≥ 500 μg/L.

At a final step, a more selective reference group (MSRG, n = 130) was defined by excluding any pregnant women with UIC < 150 μg/L, which could reflect iodine insufficiency. We emphasize that MSRG meets all NACB criteria, including sample size greater than 120 individuals.

Serum TSH, FT4, TPOAb and TgAb were performed by electrochemiluminescence immunometric assay on the Roche Modular Analytics^®^ E170 (Roche Diagnostics). The Laboratory reference ranges of TSH were 0.4 to 4.3 mIU/L (for non-pregnant women), FT4 0.7 to 1.9 ng/dL, TPOAb < 34 IU/mL and TgAb < 115 IU/mL. The intra-assay coefficients of variation of serum TSH, FT4, TPOAb and TgAb were 7.2%, 2.79%, 6.3% and 4.9%, respectively. The interassay coefficients of variation were 3%, 2.9%, 7.0% and 6.3%, respectively. Urinary iodine concentration was determined by Inductively Coupled Plasma Mass Spectrometry (ICP-MS-Spectroquant^®^ Iodine Test – Merch KGaA, Germany). The manufacturer's reference range was 26 to 705 μg/L.

All thyroid ultrasound scans were performed by a single trained examiner, using a high-frequency SIEMENS-AUSONX 300 transducer (12 MHz). Thyroid volume was calculated by the formula: length x width x thickness x 0.52 of each lobe and the isthmus. Thyroid parenchyma characteristics were described according to their echogenicity and homogeneity.

Statistical analysis was performed using the SPSS for Windows program, version 13.0. Mean, median, 2.5^th^ and 97.5^th^ percentiles were calculated for the TSH and FT4 values. Continuous variables were shown as the mean ± SD (median) and were compared between two groups using the Mann-Whitney test or Student *t*-test, according to the data distribution as evaluated by the Kolmogorov-Smirnov test. Comparisons among three or more groups were assessed by the Kruskal-Wallis test. Categorical variables were expressed as percentages and compared by the chi-squared test (χ^2^) or Fisher exact test. The analysis of the correlation between two variables was performed using Spearman's correlation coefficient (rs). A p-value < 0.05 was considered to be significant.

## RESULTS

The clinical and biochemical characteristics of the three studied groups are described in Table 1. The RG, SRG and MSRG groups were similar in terms of age, BMI, gestational week at inclusion, frequencies of smoking and iodine status. Although there were no significant differences in UIC among the studied groups, MSRG have a higher median urine iodine, classified as more than adequate status. Our study population presented normal median values of BMI when we use BMI classification tables specific to pregnant women (24).

**Table 1:** Clinical and biochemical characteristics of the three studied groups of pregnant women, evaluated in the first trimester of gestation, inhabitants a coastal area of Rio de Janeiro State

	RG (n = 225)	SRG (n = 170)	MSRG (n = 130)
Age (years)*	27.0 ± 5.0 (28.0)	27.4 ± 5.2 (28.0)	26.9 ± 5.0 (27.0)
Gestational week at inclusion (weeks)*	9.0 ± 2.0 (9.0)	9.0 ± 2.2 (9.0)	8.9 ± 2.0 (9.0)
BMI (kg/m^2^)*	25.6 ± 5.2 (24.6)	25.8 ± 5.0 (25.2)	26.1 ± 5.5 (25.2)
UIC (μg/L)*	234.0 ± 117.0 (219.0)	226.3 ± 103.6 (210.8)	278.6 ± 113.0 (252.8)
Smoking (%)	2.6 (n = 6)	3.5 (n = 6)	3.8 (n = 5)

* Mean ± standard deviation (median);

RG: Reference Group; SRG: Selective Reference Group; MSRG: More Selective Reference Group; BMI: body mass index; UIC: urine iodine concentration.

The means, 2.5^th^ percentile, 97.5^th^ percentile and median for serum TSH and FT4 values in the three studied groups are summarized in Table 2. The 97.5^th^ percentile value of TSH decreased from 4.47 mIU/L to 4.00 mIU/L after we excluded pregnant women with positive TgAb or thyroiditis pattern in ultrasound scan. In addition, median TSH and 97.5^th^ percentile value of TSH were lower in the more selective group (MSRG), which excluded any pregnant women with UIC less than 150 μg/L, compared to the other groups. In the reference group (RG), TSH was > 2.5 mIU/L in 19.6%. In the SRG and SSRG this prevalence was 17.1 and 14.6%, respectively.

**Table 2:** Reference values for serum TSH and FT4 in the first trimester of pregnancy in the three studied subgroups of pregnant women with iodine sufficiency

		TSH (mIU/L)			FT4 (ng/dL)		
	n	2.5^th^ centile	Median	97.5^th^ centile	2.5^th^ centile	Median	97.5^th^ centile
RG	225	0.12	1.33	4.47	0.80	1.10	1.50
SRG	170	0.06	1.26	4.00	0.80	1.20	1.50
MSRG	130	0.14	1.28	3.63	0.80	1.10	1.50

RG: Reference Group; SRG: Selective Reference Group; MSRG: More Selective Reference Group; TSH: thyrotropin; FT4: free thyroxine.

In the reference group (RG), TSH was slightly negatively correlated with FT4 (r_s_ −0.142; p = 0.017) but was not correlated with age (r_s_ −0.052; p = 0.221), BMI (r_s_ −0.048; p = 0.236), gestational age (r_s_ −0.025; p = 0.356) or UIC (r_s_ 0.031; p = 0.323). However, when we evaluated only the group of pregnant women up to 6 weeks of gestation, we found a negative correlation between TSH and gestational age (r_s_ −0.433; p = 0.025). This correlation was not maintained in the analysis of patients with more than 6 weeks of gestation (r_s_ 0.065 p = 0.182).

Furthermore, comparing TSH levels in these two subgroups (up to 6 weeks of gestation and > 6 weeks of gestation), we found lower levels of TSH in the group of patients up to 6 weeks of gestation. These data are described in Table 3.

**Table 3:** Reference values for serum TSH in the first trimester of pregnancy in the three studied subgroups of pregnant women according to gestational age

TSH			
	Gestational age ≤ 6 weeks	Gestational age > 6 weeks	*p* value
RG*	1.60 ± 1.32 (1.27) n = 21	1.97 ± 0.92 (1.71) n = 204	0.030
SRG*	1.47 ± 1.08 (1.24) n = 18	1.91 ± 0.89 (1.72) n = 152	0.038
MSRG*	1.41 ± 0.97 (1.26) n = 16	1.85 ± 0.93 (1.68) n = 114	0.073

* Mean ± standard deviation (median);

RG: Reference Group; SRG: Selective Reference Group; MSRG: More Selective Reference Group; TSH: thyrotropin.

## DISCUSSION

This is the first study designed to establish reference limits of serum TSH and FT4 values in a pregnant population that inhabits a coastal Brazilian area, in the state of Rio de Janeiro, that filled all NACB criteria. Additionally, to the best of our knowledge, this is the first study in the literature to apply so strict selection criteria intended to define a rational reference range of serum TSH for pregnant women in the first trimester, considering NACB criteria.

Subclinical hypothyroidism (SCH) is defined as elevated serum TSH concentrations and normal levels of serum FT4. Strategies for the diagnosis of thyroid dysfunction differ in pregnant compared to nonpregnant women.

The definition of a normal reference range of TSH in pregnant women is critical and should ideally be derived from local population data. Substantial variation exists between populations, with many recent investigations confirming a more liberal upper TSH reference range in healthy pregnant women with no thyroid disease (10).

Previously, our group accessed the distribution of serum TSH values in the first trimester of pregnancy in a group of pregnant women with negative TPOAb and iodine sufficiency in Rio de Janeiro (14). This study has also showed a 97.5^th^ percentile value of serum TSH above 2.5 mIU/L (95^th^ percentile:4.43 mIU/L – 95%CI: 3.68 to 5.84). However, the final sample number evaluated in this study, after exclusion of positive TPOAb patients, was below 120, recommended by NACB to define TSH reference range in a given population.

The disagreement between serum TSH reference values found in our study compared to those found by Rosario and cols. (11) may be due to differences between the two populations. Brazil is a continental-sized country with ethnic and geographic characteristics that differ from a region to another. The state of Rio de Janeiro is located in a coastal area, unlike Minas Gerais, which is located in a continental area. In addition, two limitations of that study were absence of iodine status evaluation and absence of ultrasound scan and TgAb measurement to exclude thyroid autoimmunity in their population.

Although some studies have shown that excluding people with thyroid ultrasound abnormalities did not affect TSH reference range (25,26), some authors suggest higher TSH levels in the presence of thyroid autoimmunity detected by both ultrasound and autoantibodies (27). It appears reasonable that, in pregnant women who exhibit immunosuppression of pregnancy, thyroid ultrasound evaluation could add information regarding prediction of autoimmune thyroid disease.

A recent study that evaluated women seeking fertility treatment showed that 5% of patients presented isolated TgAb positivity and 4% isolated TPOAb concentrations. Serum TSH values were significantly higher in the group of women with isolated positive TgAb compared to women without positive thyroid antibodies. This study demonstrated that testing for thyroid autoimmunity using only TPOAb would likely miss a small proportion of women with isolated TgAb (28).

In our study reference population, there were no cases of hypothyroxinemia. Hypothyroxinemia was excluded in the sample of pregnant women studied by the presence of normal FT4 values. We believe that FT4 is a more useful index of thyroid function during early pregnancy than is total T4. Although FT4 immunoassays have been reported to be a less accurate method during pregnancy, this interference occurs mainly in the third trimester of gestation (29,30). In addition, total T4 concentrations are highly dependent on changes in thyroxine-binding globulin (TBG) concentrations, being more variable during early pregnancy than are free T4 concentrations (31).

It should be noted that there are still some concerns about the new reference range suggested by 2017 ATA thyroid and pregnancy guidelines, since it was based on studies that evaluated few women. In the five largest studies cited, only one presented an upper limit of normal TSH above 3.5 mIU/L (32).

Although many studies suggest that subclinical hypothyroidism is associated with obstetric complications and impairment in neurocognitive development of at-risk children (33,34), the recommendations for screening and treatment remain controversial (35). The previously recommended TSH cutoff of 2.5 mIU/L appears to be too low and is likely to leading to overdiagnosis and overtreatment of thyroid disease during pregnancy (1). Additional studies evaluating the safety and benefits of treatment with levothyroxine in pregnant women with subclinical hypothyroidism are needed, especially to assess the differences between TPOAb-positive or negative patients.

In addition, the authors believe that a larger population would be desired to bring more strength to the present study, despite having met the criteria required by NACB.

In conclusion, the upper limit of serum TSH reference in the first trimester was above 2.5 mIU/L in pregnant Brazilian women that live in a coastal area of the country. The value of 3.6 mIU/L, found when iodine deficiency and thyroiditis (defined both by negative TgAb and TPOAb and normal thyroid ultrasound pattern) were excluded, appeared to match the recent ATA guidelines.

## References

[1] Alexander EK, Pearce EN, Brent GA, Brown RS, Chen H, Dosiou C, et al. 2017 Guidelines of the American Thyroid Association for the Diagnosis and Management of Thyroid Disease During Pregnancy and the Postpartum. Thyroid. 2017;27(3):315-89. 10.1089/thy.2016.0457 28056690

[2] Stagnaro-Green A, Abalovich M, Alexander E, Azizi F, Mestman J, Negro R, et al. Guidelines of the AmericanThyroid Association for the diagnosis and management of thyroid disease during pregnancy and postpartum. Thyroid. 2011;21(10):1081-125. 10.1089/thy.2011.0087 PMC3472679 21787128

[3] Laurberg P, Andersen SL, Hindersson P, Nohr EA, Olsen J. Dynamics and Predictors of Serum TSH and fT4 Reference Limits in Early Pregnancy: A Study Within the Danish National Birth Cohort. J Clin Endocrinol Metab. 2016;101(6):2484-92. 10.1210/jc.2016-1387 27115059

[4] Li C, Shan Z, Mao J, Wang W, Xie X, Zhou W, et al. Assessment of thyroid function during first-trimester pregnancy: what is the rational upper limit of serum TSH during the first trimester in Chinese pregnant women? J Clin Endocrinol Metab. 2014;99(1):73-9. 10.1210/jc.2013-1674 24276458

[5] Lambert-Messerlian G, McClain M, Haddow JE, Palomaki GE, Canick JA, Cleary-Goldman J, et al. First- and second-trimester thyroid hormone reference data in pregnant women: a FaSTER (First- and Second-Trimester Evaluation of Risk for aneuploidy) Research Consortium study. Am J Obstet Gynecol. 2008;199(1):62 e1-6. 10.1016/j.ajog.2007.12.003 PMC2529146 18585522

[6] Mannisto T, Surcel HM, Ruokonen A, Vaarasmaki M, Pouta A, Bloigu A, et al. Early pregnancy reference intervals of thyroid hormone concentrations in a thyroid antibody-negative pregnant population. Thyroid. 2011;21(3):291-8. 10.1089/thy.2010.0337 21254924

[7] Springer D, Zima T, Limanova Z. Reference intervals in evaluation of maternal thyroid function during the first trimester of pregnancy. Eur J Endocrinol. 2009;160(5):791-7. 10.1530/EJE-08-0890 19228824

[8] Bestwick JP, John R, Maina A, Guaraldo V, Joomun M, Wald NJ, et al.Thyroid stimulating hormone and free thyroxine in pregnancy: expressing concentrations as multiples of the median (MoMs). Clin Chim Acta. 2014;430:33-7. 10.1016/j.cca.2013.12.030 24380740

[9] Medici M, de Rijke YB, Peeters RP, Visser W, de Muinck Keizer-Schrama SM, Jaddoe VV, et al. Maternal early pregnancy and newborn thyroid hormone parameters: the Generation R study. J Clin Endocrinol Metab. 2012;97(2):646-52. 10.1210/jc.2011-2398 22162477

[10] Medici M, Korevaar TI, Visser WE, Visser TJ, Peeters RP.Thyroid function in pregnancy: what is normal? Clin Chem. 2015;61(5):704-13. 10.1373/clinchem.2014.236646 25829408

[11] Rosario PW, Carvalho M, Calsolari MR. TSH reference values in the first trimester of gestation and correlation between maternal TSH and obstetric and neonatal outcomes: a prospective Brazilian study. Arch Endocrinol Metab. 2016;60(4):314-8. 10.1590/2359-3997000000132 PMC10118726 26886091

[12] Lazarus J, Brown RS, Daumerie C, Hubalewska-Dydejczyk A, Negro R, Vaidya B. 2014 European thyroid association guidelines for the management of subclinical hypothyroidism in pregnancy and in children. Eur Thyroid J. 2014;3(2):76-94. 10.1159/000362597 PMC4109520 25114871

[13] De Groot L, Abalovich M, Alexander EK, Amino N, Barbour L, Cobin RH, et al. Management of thyroid dysfunction during pregnancy and postpartum: an Endocrine Society clinical practice guideline. J Clin Endocrinol Metab. 2012;97(8):2543-65. 10.1210/jc.2011-2803 22869843

[14] Felipe CL, Medina CC, Sieiro NL, Alexandru B, Mario V. Is an upper limit of 2.5 mUI/l for TSH appropriate for the first trimester of pregnancy among young TPO - women? Gynecol Endocrinol. 2010;26(1):54-7. 10.3109/09513590903159557 19657813

[15] Baloch Z, Carayon P, Conte-Devolx B, Demers LM, Feldt-Rasmussen U, Henry JF, et al. Laboratory medicine practice guidelines. Laboratory support for the diagnosis and monitoring of thyroid disease. Thyroid. 2003;13(1):3-126. 10.1089/105072503321086962 12625976

[16] Geffre A, Friedrichs K, Harr K, Concordet D, Trumel C, Braun JP. Reference values: a review. Vet Clin Pathol. 2009;38(3):288-98. 10.1111/j.1939-165X.2009.00179.x 19737162

[17] Chen L, Hu R. Thyroid autoimmunity and miscarriage: a meta-analysis. Clin Endocrinol (Oxf). 2011;74(4):513-9. 10.1111/j.1365-2265.2010.03974.x 21198746

[18] Thangaratinam S, Tan A, Knox E, Kilby MD, Franklyn J, Coomarasamy A. Association between thyroid autoantibodies and miscarriage and preterm birth: meta-analysis of evidence. BMJ. 2011;342:d2616. 10.1136/bmj.d2616 PMC3089879 21558126

[19] Pedersen OM, Aardal NP, Larssen TB, Varhaug JE, Myking O, Vik-Mo H. The value of ultrasonography in predicting autoimmune thyroid disease. Thyroid. 2000;10(3):251-9. 10.1089/thy.2000.10.251 10779140

[20] Vejbjerg P, Knudsen N, Perrild H, Laurberg P, Pedersen IB, Rasmussen LB, et al. The association between hypoechogenicity or irregular echo pattern at thyroid ultrasonography and thyroid function in the general population. Eur J Endocrinol. 2006;155(4):547-52. 10.1530/eje.1.02255 16990653

[21] Trimboli P, Rossi F, Condorelli E, Laurenti O, Ventura C, Nigri G, et al. Does normal thyroid gland by ultrasonography match with normal serum thyroid hormones and negative thyroid antibodies? Exp Clin Endocrinol Diabetes. 2010;118(9):630-2. 10.1055/s-0029-1237700 19998241

[22] Dobert N, Balzer K, Diener J, Wegscheider K, Vaupel R, Grunwald F. Thyroid sonomorphology, thyroid peroxidase antibodies and thyroid function: new epidemiological data in unselected German employees. Nuklearmedizin. 2008;47(5):194-9. 18852925

[23] Shi X, Han C, Li C, Mao J, Wang W, Xie X, et al. Optimal and safe upper limits of iodine intake for early pregnancy in iodine-sufficient regions: a cross-sectional study of 7190 pregnant women in China. J Clin Endocrinol Metab. 2015;100(4):1630-8. 10.1210/jc.2014-3704 25629356

[24] Atalah E, Castillo C, Castro R, Aldea A. [Proposal of a new standard for the nutritional assessment of pregnant women]. Rev Med Chil. 1997;125(12):1429-36. 9609018

[25] Kratzsch J, Fiedler GM, Leichtle A, Brugel M, Buchbinder S, Otto L, et al. New reference intervals for thyrotropin and thyroid hormones based on National Academy of Clinical Biochemistry criteria and regular ultrasonography of the thyroid. Clin Chem. 2005;51(8):1480-6. 10.1373/clinchem.2004.047399 15961550

[26] Hamilton TE, Davis S, Onstad L, Kopecky KJ. Thyrotropin levels in a population with no clinical, autoantibody, or ultrasonographic evidence of thyroid disease: implications for the diagnosis of subclinical hypothyroidism. J Clin Endocrinol Metab. 2008;93(4):1224-30. 10.1210/jc.2006-2300 PMC2729186 18230665

[27] Jiskra J, Bartakova J, Holinka S, Limanova Z, Springer D, Fait T, et al. Low concordance between positive antibodies to thyroperoxidase and thyroid ultrasound autoimmune pattern in pregnant women. Endocr J. 2011;58(10):849-59. 10.1507/endocrj.ej11-0026 21873803

[28] Unuane D, Velkeniers B, Anckaert E, Schiettecatte J, Tournaye H, Haentjens P, et al. Thyroglobulin autoantibodies: is there any added value in the detection of thyroid autoimmunity in women consulting for fertility treatment? Thyroid. 2013;23(8):1022-8. 10.1089/thy.2012.0562 PMC3752510 23405888

[29] Anckaert E, Poppe K, Van Uytfanghe K, Schiettecatte J, Foulon W, Thienpont LM. FT4 immunoassays may display a pattern during pregnancy similar to the equilibrium dialysis ID-LC/tandem MS candidate reference measurement procedure in spite of susceptibility towards binding protein alterations. Clin Chim Acta. 2010;411(17-18):1348-53. 10.1016/j.cca.2010.05.032 20510682

[30] Berta E, Samson L, Lenkey A, Erdei A, Cseke B, Jenei K, et al. Evaluation of the thyroid function of healthy pregnant women by five different hormone assays. Pharmazie. 2010;65(6):436-9. 20614692

[31] Korevaar TI, Chaker L, Medici M, de Rijke YB, Jaddoe VW, Steegers EA, et al. Maternal total T4 during the first half of pregnancy: physiologic aspects and the risk of adverse outcomes in comparison with free T4. Clin Endocrinol (Oxf). 2016;85(5):757-63. 10.1111/cen.13106 27187054

[32] Stagnaro-Green A. Clinical guidelines: Thyroid and pregnancy – time for universal screening? Nat Rev Endocrinol. 2017;13(4):192-4. 10.1038/nrendo.2017.17 28232666

[33] Abalovich M, Gutierrez S, Alcaraz G, Maccallini G, Garcia A, Levalle O. Overt and subclinical hypothyroidism complicating pregnancy. Thyroid. 2002;12(1):63-8. 10.1089/105072502753451986 11838732

[34] Su PY, Huang K, Hao JH, Xu YQ, Yan SQ, Li T, et al. Maternal thyroid function in the first twenty weeks of pregnancy and subsequent fetal and infant development: a prospective population-based cohort study in China. J Clin Endocrinol Metab. 2011;96(10):3234-41. 10.1210/jc.2011-0274 21832110

[35] Korevaar TIM, Medici M, Visser TJ, Peeters RP. Thyroid disease in pregnancy: new insights in diagnosis and clinical management. Nat Rev Endocrinol. 2017;13(10):610-22. 10.1038/nrendo.2017.93 28776582

